# Natural adjuvants (*PC* and *G2*) induce activated natural killer cells with NKG2D expression and cytotoxic properties in colorectal cancer 

**Published:** 2022

**Authors:** Ali khodadadi, Abdolhassan Talaiezadeh, Yuji Heike, Hamid Galehdari, Mojtaba Oraki Kohshour, Abdolkarim Sheikhi, Seyed Nematollah Jazayeri, Mohammad Pedram, Mehrdad Borhani, Ali Asadirad

**Affiliations:** 1 *Department of Immunology, Faculty of Medicine, Ahvaz Jundishapur University of Medical Sciences, Ahvaz, Iran.*; 2 *Cancer, Petroleum and Environmental Pollutants Research Center, Ahvaz Jundishapur University of Medical Sciences, Ahvaz, Iran. *; 3 *Department of Surgery, Faculty of Medicine, Ahvaz Jundishapur University of Medical Sciences, Ahvaz, Iran.*; 4 *Department of Hematopoietic Stem Cell Transplantation, National Cancer Centre Research and Development, Tokyo, Japan.*; 5 *Department of Genetics, Faculty of Science, Shahid Chamran University of Ahvaz, Iran. *; 6 *Department of Immunology, Faculty of Medicine, Dezful Faculty of Medical Sciences, Dezful, Iran.*; 7 *Thalassemia & Hemoglobinopathy Research center, Health research institute, Ahvaz Jundishapur University of Medical Sciences, Ahvaz, Iran*; 8 *Department of Statistics, Faculty of Epidemiology, Ahvaz Jundishapur University of Medical Sciences, Ahvaz, Iran*; † *Deceased*

**Keywords:** Colorectal cancer, Natural killer cells, PC adjuvant, G2 adjuvant, NKG2D, Cytotoxicity

## Abstract

**Aim:**

This study aimed to investigate the effects of natural adjuvants (*G2 *and *PC*) to activate natural killer cells in colorectal cancer.

**Background:**

Natural killer (NK) cells are an element of the innate immune system that can recognize and kill cancer cells and provide hope for cancer therapy. One of the current methods in cancer immunotherapy is NK cell therapy. Immunotherapy with NK cells has been limited because of the low number and cytotoxicity level of NK cells. Natural adjuvants such as *PC *and* G2* may stimulate the immune system. It seems that these adjuvants could increase cytotoxic NK cells.

**Methods:**

Twelve patients with colorectal cancer and six healthy individuals qualified for inclusion in this study. Peripheral blood mononuclear cells (PBMCs) from each patient with two distinctive concentrations (10^5^and 5×10^4^ cells/well) were treated with Interleukin2 (IL2), *PC*, and *G2* adjuvant separately. The NK cell's surface markers, including CD16, CD56, and NKG2D, were evaluated by flow cytometry. The cytotoxicity effect of treated PBMCs as effector cells against NK sensitive cell line (K562) was assessed using the LDH assay method.

**Results:**

The results revealed a significant increase in the level of CD16+NKG2D+ NK cells in PBMCs treated with the *G2* group compared with the control group in CRC PBMC (*p*<0.001) as well as the normal PBMC group (*p* < 0.01). In addition, the results indicated a significant increase in the level of CD56+NKG2D+ cells in the PBMC treated with *PC* (*p* < 0.05) and *G2* (*p* < 0.001) groups compared with the PBMC group. The cytotoxicity result of PBMC from CRC patients in 10:1 ratio of the effector: target showed that the cells^'^ cytotoxicity in the PBMCs treated with *PC* (*p*<0.01) and *G2* (*p*<0.05) was significantly higher than the untreated PBMC.

**Conclusion:**

According to the result of this study, it can be stated that the *PC* and *G2* adjuvants could be candidates for inducing cytotoxic natural killer cells.

## Introduction

 Colorectal (CRC) cancer is the third most commonly diagnosed tumor in the world. As nutrition and lifestyles have changed in the past decade, the cases of colon cancer and other gastrointestinal (GI) cancers have increased quickly in the Eastern world. While surgical operation is the most common treatment option, this cancer is treated efficiently with chemotherapy, radiotherapy, immunotherapy, or combination therapy to increase survival (1, 2). Several therapeutic plans for metastatic CRC cancer have been considered in recent years, but most patients in the progressive stages have low expectations for prolonged survival. Therefore, the current therapeutic method for patients who have CRC cancer must be improved. Recently, much attention has been paid to cancer immunotherapy to control metastatic disease, decrease the recurrence rate, increase the recurrence time, and eventually, as a preventive action (3). The concept of adaptive immunotherapy, which involves transferring immune cells with anti-tumor activities to the tumor-bearing host to mediate the regression of established primary and metastatic neoplasm, has been successfully employed in animal models (4). Recently, the efficiency of immunotherapy in the treatment of several types of cancer has been considered. For example, tumor-reactive T helper 1(TH1) and TH17 cells as adoptive cells transferred in melanoma cancer mediated anti-tumor immunity and led to melanoma deterioration (5). Among immunologic effector cells, NK cells are the best choice for immunotherapy, because they have mediators for anti-tumor effects. NK cells differentiate from CD34+ hematopoietic stem cells. They are known as an element of the innate immune system that recognize and kill cancer cells. These cells are regulated with inhibitory and stimulatory signals to attack cancer cells without sensitization (6, 7). NKG2D activates NK receptors that bind to MIC-A, MIC-B, and ULBPs (MHC class I-like ligands) on cancer cells. After this connection, the NK cells are activated. Activated NK cells consequently increase the expression of the NKG2D receptor and cytotoxicity against cancer (8, 9).

NK cells usually present with low numbers in PBMCs. To improve the efficiency of NK cells, lymphokine-activated killer (LAK) and cytokine-induced killer (CIK) cells were prepared in vitro (10-12). Prolonged cultures of PBMC with IL2 significantly expand CIK cells, and the addition of IFN-γ improves the cytotoxicity of CIK cells (13).

More than half of CRC patients die due to cancer-associated complications. Therefore, the development of immunotherapy, including adjuvant therapy and other alternative therapies, is crucial. Cancer immunotherapy with NK cells activated with IL2 has been successful, especially for patients with leukemia (14, 15); however, the adoptive transfer of interleukin IL2‐activated NK cells in solid tumors did not lead to acceptable results (16). Moreover, treatment with the first drug did not lead to satisfactory results.

*G2* adjuvant is an activator vaccine for the immune system registered as a patent at the Iranian Patent Office (Innovation Register No: 36679, 28 October 2006). The *G2* adjuvant produces buffalo spleen lipids and consists of diverse lipids, cholesterol, glucose, and triglycerides (17). 

*PC* adjuvant is also known as an activator vaccine for the immune system and is registered as a patent at the Iranian Patent Office (Innovation Register No: 36681, 28 October 2006). The *PC* adjuvant is produced from the bacterial extract polypeptide comprised of proteins, polypeptides, phospholipids, and polysaccharides (17).

The efficacy of the* G2* and *PC* adjuvants was determined in animal models of asthma and breast cancer. These adjuvants stimulate TH1 cells, prevent IL4 production, and change naive T cells to TH2 cells (19). They also increase IFNγ levels (18), which are synergistic with IL2, to promote CIK cell differentiation in PBMCs.

A previous study has shown that the *G2 *and *PC *adjuvants could stimulate PBMCs. New strategies can be developed for the *ex vivo* enhancement of the antitumor activity and cytotoxicity of NK cells intended for transfer into patients with solid tumors. To the best of our knowledge, this is the first study to examine the effect of *PC* and *G2* adjuvants on CRC and normal PBMCs. The innovation of this work is the usage of natural adjuvants to activate NK cells to improve the cytotoxicity and performance of immunotherapy. Thus, we aim to explore the ability of *PC *and *G2* to expand cytotoxic NK cells in CRC patients. 

## Methods

**Patients**

Between July 2019 and July 2020, CRC patients from Imam Khomeini Hospital, Ahvaz, Iran, who met the inclusion criteria were considered suitable participants in the current study. The indication for a screening colonoscopy or follow-up endoscopy after polypectomy, surgery, or pathological examination was included. Patients with familial adenomatous polyposis or hereditary nonpolyposis CRC, intraepithelial neoplasia, any other malignancy, or acute gastrointestinal bleeding as well as the inability to provide informed consent were excluded. 

The protocol was explained and written informed consent for participation in the study obtained from each patient who entered this study. The protocols were accepted by the Ethics Committee of Ahvaz Jundishapur University of Medical Sciences. In all, 12 patients and 6 healthy donors fulfilled the eligibility criteria. 

**PBMC Separation**

Blood sampling was performed on the morning of the operation day before any treatment. PBMCs were obtained with *Ficoll-hypaque* centrifuging technique, then cultured in RPMI 1640 medium containing pen strep (150 µg/ml). Amphotericin-B (2 µg/ml) PBMCs of each patient with concentrations of $10^{5}$ and 5 × $10^{4}$ were allocated in 96-well micro-plates in triplicate (20, 21).

**PBMC activation with IL**_2_

PBMCs were treated with interleukin_2_ (250 IU/ml) (*Santa Cruz Biotechnology*, SC/4593/lot l16.9) then incubated overnight to generate LAK cells. Following the primary dose-response studies of lymphocyte proliferation and LAK cell generation, IL_2_ concentration was selected (22, 23).

**PBMC Activation with** **adjuvant**

The *PC* adjuvant was prepared from the bacterial-polypeptide extract. To evaluate its effect on PBMCs, 2 µg/ml of the *PC* adjuvant was added to the PBMCs and then incubated overnight. This concentration was selected as previously described (15, 16).

The *G2* adjuvant was prepared from buffalo spleen lipids. To evaluate this adjuvant's effect on PBMCs, *G2* adjuvant (10 µg/ml) was added to PBMCs and then incubated overnight. This concentration was selected as previously explained (15, 17).

**Evaluation of NK cells surface marker**

To assess the effects of *PC* and *G2* on NK cells, PBMCs were treated with IL2, *PC*, and *G2* overnight. NK cells surface markers comprising CD3-APC (Bio Legend, CA, USA), CD16-FITC (Bio Legend, CA, USA), CD56-PE (Bio Legend, CA, USA), and NKG2D-PE (Bio Legend, CA, USA) were evaluated with flow cytometry in the following groups: PBMCs without any treatment (PBMC), PBMCs treated with 250 IU/ml of interleukin2 (PBMC+IL2), PBMCs treated with 2 µg/ml of *PC* (PBMC+*PC*), and PBMCs treated with 10 µg/ml of *G2* (PBMC+*G2*). After NK cells were labeled with the antibodies for 30 minutes at 4 °C, they were washed twice with washing buffer and then detected by flow cytometry (BD, CA, USA) (24). The data analysis was done by FlowJo software version 7.6.

**Cytotoxicity test**

The cytotoxicity of cytokine and adjuvant activated cells as effector cells were evaluated against the K562 cell line as target cells. The natural killing ability of NK cells was measured using a cytotoxicity detection kit. Cell cytotoxicity was detected by measuring LDH that leaked out of damaged cells. The experiment was carried out in a 96-well micro-plate in triplicate. The PBMCs of each patient as effector cells were cultured with $10^{5}$ and 5 × $10^{4}$ cells/well. LDH assay was done in the PBMC, PBMC+IL2, PBMC+PC, and PBMC+*G2* groups. Then, the micro-plates were set in the incubator overnight to generate cytotoxic natural killer cells.

Next, the NK sensitive cell line K562 was added to each well as the target cells (10^4^cells/well) for 4 hours to evaluate the cytotoxicity effect of effector cells. In this phase, the effector cell's cytotoxic activity led to leakage of lactate dehydrogenase (LDH) from the target cell into the culture medium. Therefore, by measuring the LDH value, the cytotoxic activity was determined. The LDH value was determined using a Cytotoxicity Detection Kit^PLUS^ (LDH) from Roche Applied Science (Roche Diagnostics Deutschland, GmbH, Sand Hofer, Cat. Number 4744926001). 

To detect LDH values, the substrate mixture (available in the kit) was added to each well. Any LDH leakage during the previous step could reduce the tetrazolium salt into formazan by a coupled enzymatic reaction. Thus, the release of LDH from the target cells was directly correlated with the amount of formazan formed in this step. Then, the formazan was quantitated with an ELISA plate reader. The formazan dye was water-soluble and showed a maximum absorption rate at about 500 nM (25).

Cytotoxicity percentage of the effector cells was c according to the following conventional formula (26):

$\mathrm{Cytotoxicity}(\%)=\frac{(\mathrm{Effector}:\mathrm{Test Cell Mix}-\mathrm{Effector Cell Control})-\mathrm{Low Control}}{\mathrm{High Control}-\mathrm{Low Control}}\times \mathrm{100}$

In this formula, low control indicates the LDH activity released from the untreated target cells and contains assay medium and target cells. High control indicates the highest level of releasable LDH activity from the target cells and contains triton x 100 solution and target cells.

**Statistical analysis**

Experimental results are presented as mean± standard error for the considered groups. For data normalization, logarithmic transformation was done based on the data distribution. Two-way analyses of variance (ANOVA) and Graph Pad Prism 4.0 software were used for statistical analysis. Results were considered statistically significant when *p*-values < 0.05. 

## Results

**NK cells surface marker**

**Figure 1:**
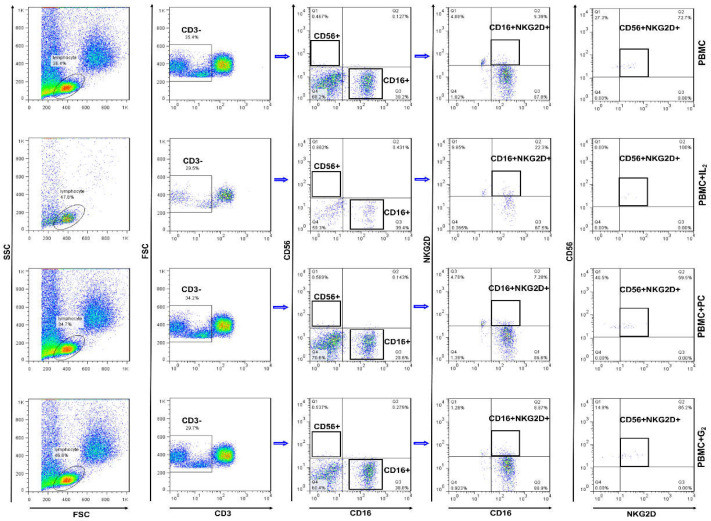
Dot plot diagrams of NK surface marker in different groups. Analysis of surface markers (CD3-APC, CD16-FITC, CD56-PE, and NKG2D-PE) in different groups including PBMC without any treatment** (A)**, PBMC treated with 250 IU/ml of Interleukin2 **(B)**, PBMC treated with 2 µg/ml of *PC* **(C)**, and PBMC treated with, 10 µg/ml of *G2*** (D)** was done by flow cytometry. Profiles demonstrate the gating strategy for the NK cells subset create a “lymphocyte gate” on a forward scatter (FCS) vs. side scatter (SSC) plot. After that, in the FSC vs. CD3 plot, CD3^−^ cells were gated; a plot with CD56 vs. CD16 can be used to identify the main NK cell subsets. Then cells with CD16+ NKG2D+ and CD56+ NKG2D+ were gated

To investigate the impact of the IL2, *PC*, and *G2* adjuvants on NK cells in normal and CRC PBMC, NK cells surface markers (CD3-, CD16+, CD56+, and NKG2D+) were measured in the PBMC, PBMC+IL2, PBMC+*PC*, PBMC+*G2* groups (Figure 1).

**Figure 2:**
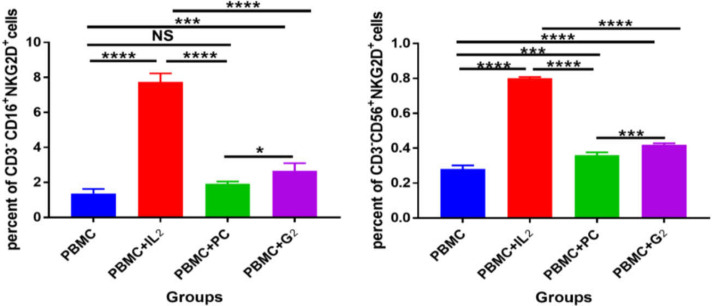
The mean ± SD triplicate percentage of CD3-CD16+NKG2D+ cells (A) and percentage of CD3-CD16+NKG2D+ cells (B) in different groups. The percentage of CD3-CD16+NKG2D+ (A) and CD3-CD16+NKG2D+(B) cells in different groups including PBMC without any treatment (PBMC), PBMC treated with 250 IU/ml of Interleukin2 (PBMC+IL2), PBMC treated with 2 µg/ml of *PC* (PBMC+*PC*), and PBMC treated with 10 µg/ml of *G2* (PBMC+*G2*) in normal and CRC PBMCs were done by flow cytometry. The results indicate that the PC adjuvant can significantly increase CD56+NKG2D+ cells in CRC and normal PBMCs. However, the *G2* can significantly increase CD16+NKG2D+ and CD56+NKG2D+ cells in CRC PBMCs and normal PBMCs. Statistical analysis was performed using two-way ANOVA (NS: not significant, * *p* < 0.05, ** *p* < 0. 01, *** *p* < 0.001).

Analysis of flow cytometry data showed that the percentage of CD16+NKG2D+ cells was significantly (*p*<0.001) higher in PBMCs treated with IL2 than in other groups. This result was similar in both CRC and normal PBMCs. It was also shown that in the PBMC, PBMC+*PC*, and PBMC+*G2* groups, the percentage of CD16+NKG2D+ cells in the CRC PBMCs compared with the normal PBMC was not significant (*p *> 0.05). The results demonstrated no significant difference in the percentage of CD16+NKG2D+ cells in the PBMC+*PC* group compared with the PBMC group in both CRC and normal PBMCs. The results revealed a significant increase in the level of CD16+NKG2D+ cells in the PBMC+*G2* group compared with the PBMC group in CRC PBMCs (*p *< 0.001) as well as normal PBMCs (*p* < 0.01). Analysis of the results showed significant differences in the percentage of CD16+NKG2D+ cells in the PBMC+*PC* and the PBMC+*G2* groups compared with the PBMC+IL2 group in CRC and normal PBMCs (Figure 2A).

Analysis of flow cytometry data in all groups showed that the percentage of CD56+NKG2D+ cells was significantly higher in CRC PBMCs than in normal PBMCs (*p *< 0.001). The results further demonstrated that PBMCs treated with the IL2 in the PBMC+IL2 group had a significantly higher amount of CD56+NKG2D+ cells compared with the other groups (*p* < 0.001). The percentage of CD56+NKG2D+ cells in the PBMC+IL2 group in CRC PBMCs was significantly higher than in normal PBMCs (*p *< 0.001). The results revealed a significant increase (*p* < 0.05) in the level of CD56+NKG2D+ cells in the PBMC+PC group compared with the PBMC group. The percentage of CD56+NKG2D+ cells in the PBMC+*G2* group was also increased significantly (*p* < 0.001) compared with the PBMC group (Figure 2B). 

The current study has shown that the *PC* adjuvant can significantly increase the level of CD56+NKG2D+ cells in both CRC and normal PBMCs. However, *G2* can significantly increase the CD16+NKG2D+ and CD56+NKG2D+ cells in both CRC PBMCs and normal PBMCs.

**Analysis of the cytotoxicity test**

**Figure 3:**
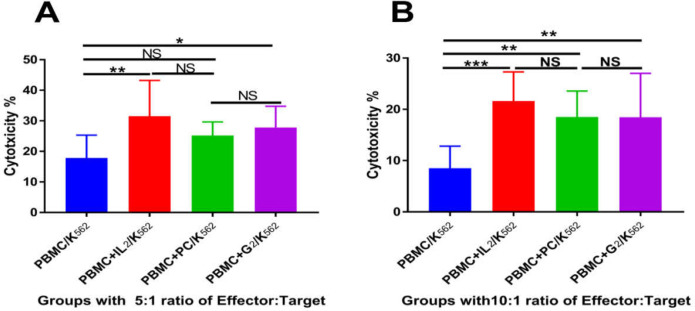
The mean ± SD triplicate percentage of the cytotoxicity test with 5:1 (A) and 10:1 (B) ratios of Effector (Normal and CRC PBMC): Target (K562 cells). Normal and CRC PBMCs in different groups including PBMC without any treatment (PBMC), PBMC treated with 250 IU/ml of Interleukin2 (PBMC+IL2), PBMC treated with 2 µg/ml of *PC* (PBMC+*PC*), and PBMC treated with 10 µg/ml of *G2* (PBMC+*G2*) were co-cultured with the K562 cell line as NK sensitive cells. Then, cytotoxicity against K562 cells was detected by the LDH assay method. The result of 5:1 ratio showed that the *G2* adjuvant can activate the NK cells; it can also induce cytotoxic NK cells in CRC PBMCs better than in normal PBMCs (*p* < 0.001). The results in the 10:1 ratio indicate that the PC and *G2* adjuvants can activate the NK cells in CRC PBMCs better than in normal PBMCs (*p* < 0.001). In addition, the results showed that the *PC* adjuvant could activate NK cells as well as IL2. Statistical analysis was performed using two-way ANOVA (NS: not significant, * *p* < 0.05, ** *p* < 0.01, *** *p* < 0.001).

A cytotoxicity test was done to evaluate the cytotoxicity of adjuvant activated NK cells as effector cells against the K562 cells as target cells. For the cytotoxicity test, PBMCs from CRC patients and normal individuals in the PBMC, PBMC+IL2, PBMC+*PC*, and PBMC+*G2* groups were co-cultured with K562 cells in effector-to-target cells ratios of 5:1 and 10:1 (Figure 3).

**Analysis of cytotoxicity data in an effector to target cells ratio 5:1**

PBMCs from normal individuals showed that the cytotoxicity effect of activated NK cells in the PBMC+IL2 (*p *< 0.01) and PBMC+*G2 *(*p *< 0.05) groups was significantly higher than in the PBMC group. In addition, the difference in cell cytotoxicity between the PBMC+PC group and the PBMC group was not significant. The results revealed a significant increase (*p* < 0.01) in cell cytotoxicity in the PBMC+IL2 group compared with the PBMC+G2 group. 

Moreover, the cytotoxicity results showed that the difference in cell cytotoxicity between the PBMC+*PC* and the PBMC+*G2* groups was not statistically significant.

The results of CRC PBMCs showed that cell cytotoxicity was significantly higher in the PBMC+IL2 (*p *< 0.001) and PBMC+*PC* (*p *< 0.05) groups than the PBMC group. In contrast, the results demonstrated no significant difference in the percentage of cytotoxicity effect of cells in the PBMC+*G2* group compared with the PBMC group. Moreover, the difference in cell cytotoxicity between the PBMC+*PC* group and the PBMC+ IL2 group was not significant. The results further revealed that cell cytotoxicity was significantly higher in the PBMC+IL2 (*p *< 0.05) group than the PBMC+*G2* group.

The difference in cytotoxicity effects of NK cells in the PBMC+*G2* group between CRC PBMCs and normal PBMCs was significant (*p *< 0.001). In contrast, there was no significant difference between CRC and normal PBMCs in the PBMC, PBMC+IL2, or PBMC+*PC* groups. This result revealed that the *G2* adjuvant could activate the NK cells in CRC patients better than in normal PBMCs (Figure 3A).

**Analysis of cytotoxicity data in an effector-to-target cells ratio of 10:1**

PBMCs from normal individuals showed that the cytotoxicity effect of activated NK cells was significantly higher in the PBMC+IL2 (*p *< 0.05) group than the PBMC group. In addition, the differences in cell cytotoxicity between the PBMC+*PC* and PBMC+*G2* groups and the PBMC group were not significant. The results revealed a significant increase (*p* < 0.01) in cell cytotoxicity in the PBMC+IL2 group compared with the PBMC+*G2* group. Moreover, the cytotoxicity results showed that the difference between the cytotoxicity of cells in the PBMC+*PC* and the PBMC+*G2* groups was not statistically significant.

The result of PBMCs from CRC patients showed that cell cytotoxicity in the PBMC+IL2 (*p *< 0.001), PBMC+*PC* (*p *< 0.01) and PBMC+*G2 *(*p *< 0.05) groups was significantly higher than in the PBMC group. Moreover, the difference in cell cytotoxicity between the PBMC+*PC* and the PBMC+ IL2 groups was not significant. The results revealed that cell cytotoxicity was significantly higher in the PBMC+IL2 (*p *< 0.05) group than in the PBMC+*G2* group.

Analysis of cytotoxicity data in the 10:1 ratio of the effector: target cells showed that the cytotoxicity of activated NK cells was significantly higher in CRC PBMCs than in normal PBMCs in all groups, i.e. the PBMC (*p *< 0.05), PBMC+IL2 (*p *< 0.01), PBMC+*PC* (*p *< 0.001), and PBMC+*G2 *(*p *< 0.001) groups. This result revealed that *PC* and *G2* adjuvants could activate NK cells in CRC patients better than normal PBMCs in this ratio. In addition, the results indicated that the *PC* adjuvant could activate NK cells as well as IL2 (Figure 3B).

## Discussion

The current study has shown that *PC* and *G2* adjuvants can expand cytotoxic natural killer cells. NK cell expansion was observed in all PBMCs tested, including those of patients with colorectal cancer and those of normal individuals. This work presents a new method for *in vitro* expansion of cytotoxic NK cells with natural adjuvants. To the best of our knowledge, this is the first study to investigate the effects of *PC* and *G2* adjuvants on CRC and normal PBMCs to expand cytotoxic NK cells. The results demonstrate that IL2 can increase the percentage of NK cells with CD16+NKG2D+ and CD56+ NKG2D+ phenotypes in PBMCs. NKG2D is the stimulatory receptor of NK cells that bind to MIC-A, MIC-B, and ULBPs, which are highly up-regulated in many tumor cell types. After this connection, the NK cells are activated. Activated NK cells consequently increase the expression of the NKG2D receptor and the cytotoxicity against cancer (9). This study also revealed a simultaneous increase in the expiration of CD16 and NKG2D markers on NK cells with the cytotoxic effect of NK cells. The percentage of CD56+ NK cells in PBMCs activated with IL2 increased significantly. The CD56+ NK cell is considered an efficient cytokine producer cell with immunoregulatory properties; however, it can also become cytotoxic upon appropriate activation, like IL2-activated NK cells (27). Previous studies confirm the increasing cytotoxicity effect of NK cells after IL2 stimulation (14, 28).

The current study has shown that the *PC* adjuvant increased the percentage of CD56+ NKG2D+ cells and the cytotoxic properties of NK cells in CRC and normal PBMCs. A previous study demonstrated that the *PC* adjuvant has stimulatory properties for TH1 and NK cells with IFNγ production (17, 18). Some studies have indicated that both subsets of NK cells, CD16+ and CD56+, have cytotoxic properties and can kill tumor cells. Although CD16+ NK cells are typically cited as being the more cytotoxic subset due to higher perforin, granzymes, and cytolytic granules (29), other studies have shown that following activation, CD56+ NK cells are equally, if not more, cytotoxic (30, 31). The current study confirms that following *PC* activation, the CD56+ subset of NK cells and cytotoxic effect of NK cells were increased significantly.

The results further revealed that the *G2* adjuvant increased the percentage of CD56+ NKG2D+ and CD16+NKG2D+ cells and increased the cytotoxic properties of NK cells in CRC and normal PBMCs. The *G2* adjuvant has stimulatory properties for TH1 cells and prevents naive T cells from changing to TH2 cells to prevent IL4 production (17, 18). This adjuvant can prevent the increase of eosinophils and basophils and control the allergic response, following inhibition of TH2-related responses (32, 33). A previous study showed that *G2* adjuvant therapy gradually shrunk the tumor mass until it disappeared completely (34). This adjuvant can also contribute to cancer combination therapy by activating immune cells such as NK cells (19, 34). Another study confirms the present results, indicating that the *G2* adjuvant can persuade activated killer cells (32,35).

The current results revealed that the percentage of total NK cells was increased in patients with CRC. Another study has confirmed this result (36). It has also shown that the percentage of NKG2D, NKp30, NKp46, and perforin positive NK cells was significantly down-regulated in CRC; reduced levels of these molecules were associated with indicators of disease progression (36). The reduced level of stimulatory markers with an increasing percentage of NK cells causes the inefficiency of NK cells. Natural adjuvants could activate NK cells and increase their cytotoxicity properties.

The current study demonstrated that the *PC* and *G2* adjuvants could induce cytotoxic natural killer cells. However, the ability of these adjuvants to induce cytotoxic natural killer cells was less than that of IL2. Cytotoxic killer cells induced by the *PC* and *G2* adjuvants due to NKG2D receptor expression could detect tumor cells with high performance. They are excellent options to improve the antitumor response in CRC and other metastatic cancers.

In conclusion, the present study has demonstrated that *PC* and *G2* adjuvants can induce cytotoxic natural killer cells with NKG2D receptor expression. They can also induce cytotoxic NK cells with significant cytotoxicity properties. Finally, it can be stated that *PC* and *G2* adjuvants can be an option to induce cytotoxic killer cells. These activated cells could kill tumor cells and increase antitumor activity in solid tumors. Nevertheless, for clinical application, further investigations and *in vivo* studies are required.

## Conflict of interests

The authors declare that they have no conflict of interest.
